# Psilocybin Treatment as an Adjunct to Cognitive Behavioral Therapy for Eating Disorders: Therapeutic Rationale & Considerations for Protocol Development

**DOI:** 10.3390/bs16030376

**Published:** 2026-03-06

**Authors:** Elena Koning, Susan Gamberg, Aaron Keshen

**Affiliations:** 1 Department of Psychiatry, Dalhousie University, Halifax, NS B3H 2E2, Canada; susan.gamberg@nshealth.ca (S.G.); aaron.keshen@nshealth.ca (A.K.); 2 Eating Disorders Program, Nova Scotia Health, Halifax, NS B3H 2E2, Canada

**Keywords:** psilocybin, eating disorders, psychotherapy, psychedelic treatment

## Abstract

Eating disorders (EDs) remain challenging to treat, with high dropout and low remission rates in cognitive-behavioral therapy for EDs (CBT-ED). Psilocybin treatment (PT) demonstrates therapeutic potential to enhance CBT-ED by exerting several neurobiological, psychological, and experiential effects (e.g., antidepressant, neuroplasticity, emotional openness) that are hypothesized to increase psychotherapeutic engagement, reduce dropout, and improve clinical outcomes. This narrative review provides the first consolidation of theoretical evidence for PT/CBT-ED, proposes considerations for a concurrent intervention protocol, and presents clinical and research considerations to empirically test its feasibility, safety, and efficacy. This line of inquiry is expected to advance the development of approaches that improve ED treatment outcomes and, more broadly, advance the study of psychedelics as tools to enhance evidence-based psychotherapy models.

## 1. Introduction

Eating disorders (EDs) are a group of severe psychiatric conditions that negatively impact quality of life and daily functioning in ~1–3% of the global population (Qian et al., 2022; Shen et al., 2025; Smink et al., 2012). Among the most frequently studied EDs, anorexia nervosa (AN), bulimia nervosa (BN), and binge eating disorder (BED) are characterized by maladaptive eating behaviors, neurocognitive dysfunction, and psychological distress, contributing to an increased risk of potentially fatal medical and psychiatric complications (Amianto et al., 2015; Castillo & Weiselberg, 2017; Foldi & Griffiths, 2025; Harrington et al., 2015; van Eeden et al., 2021; van Hoeken & Hoek, 2020). The leading evidence-based treatment for EDs is cognitive-behavioral therapy (CBT-ED), developed to alter the cognitive and behavioral processes that maintain ED psychopathology (e.g., cognitive distortions about body shape and weight and dietary restraint) (Fairburn, 2008a; Fairburn et al., 2009; Murphy et al., 2010). However, sustained recovery following CBT-ED is only achieved in 40–50% of patients. Approximately 25–50% of patients dropout from CBT-ED prematurely, representing a major barrier to treatment success (Linardon, 2018; Linardon et al., 2018; Pinna et al., 2014). 

The reason for suboptimal CBT-ED outcomes is not fully known and likely involves several neurobiological and psychological mechanisms associated with EDs and psychotherapeutic engagement (Coker et al., 1993; Fassino et al., 2009; Halmi, 2013; Steel et al., 2000; Vall & Wade, 2015). Dropout is associated with a lack of early symptom improvement, depressive symptoms, and experiential avoidance (Agras et al., 2000; Dixon et al., 2025; Fassino et al., 2002, 2009; Steel et al., 2000; Wagner et al., 2015). Pharmacotherapy can target mechanisms underlying suboptimal treatment response and premature dropout and has been shown to enhance outcomes when combined with psychotherapy for EDs (Grilo et al., 2024; Walsh et al., 1997). However, fluoxetine for BN and lisdexamfetamine for BED are the only pharmacological treatments approved for EDs, and, to our knowledge, no medications have demonstrated efficacy in significantly reducing dropout from CBT-ED (Crone et al., 2023; Fluoxetine Bulimia Nervosa Collaborative Study Group, 1992; Rodan et al., 2023). There is a critical need to develop innovative approaches that enhance psychotherapeutic engagement and reduce dropout in ED clinical care.

Psilocybin treatment (PT) is emerging as a therapeutic approach in psychiatry, including robust antidepressant effects upon meta-analysis (Fang et al., 2024; Haikazian et al., 2023; Rosenblat et al., 2023) and reduced ED psychopathology in several small, open-label trials (Bevione et al., 2025; Gukasyan et al., 2022; Koning & Brietzke, 2023; Peck et al., 2023; Zhu et al., 2025). PT involves the administration of psilocybin—a plant alkaloid derived from the *Psilocybe* mushroom—in a medically supervised and psychologically supported context (Barber & Aaronson, 2022; Nichols, 2016). While PT most often occurs with general psychological support, there is growing scientific interest for its utility as a tool to enhance structured psychotherapies, supported by theoretical and empirical lines of evidence (Goodwin et al., 2024; Monson et al., 2020; Sloshower et al., 2023; Wolff et al., 2025). For example, PT exerts several acute/subacute neurobiological (e.g., serotonergic, neuroplastic) and psychological (e.g., antidepressant, cognitive flexibility, experiential openness) effects that may be conducive to psychotherapeutic engagement and efficacy (Doss et al., 2021; MacLean et al., 2011; Mertens et al., 2020; Zhao et al., 2024). Therefore, leveraging PT to enhance evidence-based psychotherapy is considered a highly promising direction by both ED patients and experts in the psychedelic literature, but no study has investigated PT as a tool to enhance CBT-ED (Cuerva et al., 2024; Goodwin et al., 2024; Wolff et al., 2025). 

The standard PT model is largely derived from historical research practices, involving non-specific, non-directive psychological support throughout preparation, dosing and integration sessions (Brennan & Belser, 2022; Cavarra et al., 2022; Kelly et al., 2021). However, transdiagnostic evidence and preliminary trials in EDs suggest that the addition of structured, evidence-based psychotherapy alongside PT may be necessary for improved and sustained behavioral changes (Johnson et al., 2017; Peck et al., 2023). Moreover, experts in the psychedelic literature call for the development of standardized psychotherapy protocols to improve reporting, measure fidelity, and promote replicability (Brennan & Belser, 2022; Cavarra et al., 2022; Chisamore et al., 2024; Hultgren et al., 2025). No publications have formally consolidated the theoretical rationale or developed a combined PT/CBT-ED protocol. Therefore, the current paper aims to do so by proposing PT/CBT-ED protocol considerations, specifically for non-underweight EDs (i.e., BED and BN), contextualized by its therapeutic rationale and implications for future research to test its feasibility, safety, and efficacy in ED treatment.

## 2. Methods

To characterize the theoretical rationale for PT/CBT-ED, a literature search was conducted to identify theoretical and empirical evidence for an effect of psilocybin treatment on CBT retention and outcomes in EDs. The online databases PubMed, PsycINFO, and Embase were searched for relevant publications from inception to September 2025 using a combination of the terms: psilocybin, psychotherapy, eating disorders, bulimia, anorexia, binge eating, body image, depressive symptoms, neuroplasticity, dropout, engagement, therapeutic alliance, cognitive/psychological flexibility, social function, and psychedelic experience. Additional sources were identified via bibliographic search inspection and targeted search. The articles included in this narrative review were not selected systematically and cannot be classified as all-inclusive. 

To develop considerations for the development of the PT/CBT-ED protocol, the authors consulted established CBT-ED and psychedelic therapy manuals (e.g., MAPS Manual for MDMA-Assisted Psychotherapy, Yale Manual for Psilocybin-Assisted Therapy), and relevant scientific literature specific to ED psychotherapy (e.g., Russell et al., 2023). As extensive work has already been done to develop psychedelic psychotherapy frameworks, the focus of protocol considerations prioritized modifications of the traditional PT model that may be beneficial to participants with EDs, to reduce anticipated risks and enhance any therapeutic effects. The following questions guided the development of protocol considerations: (1) Who should conduct PT and CBT-ED sessions and what formal training should be required to do so?; (2) How many PT sessions (i.e., preparation, dosing, integration) should be conducted alongside CBT-ED and when should they be conducted?; (3) In the context of an ED, what content should be included in the preparation session(s)?; (4) What are important considerations for psychological and medical support during the dosing session?; (5) In the context of an ED, what should be included in the integration session(s), including linking session content to CBT-ED concepts?; and (6) Are there any other important considerations to address in the development of a PT/CBT-ED protocol?

## 3. Theoretical Rationale for PT/CBT-ED

The hypothesis that PT will augment the effects of CBT-ED (e.g., improve dropout rates and enhance clinical outcomes) represents a logically valid argument that is testable, falsifiable and clear (Thompson & Skau, 2023). Although never empirically tested, the potential additive therapeutic effect of PT and CBT-ED is supported by several converging empirical and theoretical findings (Fairburn et al., 2009; Öst et al., 2024; Evens et al., 2023; Majić et al., 2015; Sampedro et al., 2017). The specific neurobiological and psychological mechanisms underlying this rationale and supporting the primary hypothesis introduced herein are described in the following sections and summarized in Table 1.

### 3.1. Neurobiological Effects

#### 3.1.1. Serotonergic Neurotransmission

Psilocybin exerts several neurobiological effects that are hypothesized to augment the effects of CBT-ED, demonstrated by several lines of preclinical and clinical research. Psilocybin is often referred to as a serotonergic psychedelic, a classification that reflects the similarities in chemical structure between psilocybin and the neurotransmitter serotonin (Adebo et al., 2025; Nichols, 2016). As such, the agonistic effects of psilocin—the active metabolite of psilocybin—at serotonin receptors is well-documented, indicating a mechanism for increased serotonergic neurotransmitter signaling following psilocybin administration (Adebo et al., 2025; Madsen et al., 2019; Wulff et al., 2023). Serotonin is crucially implicated in eating behavior, cognition and mood regulation (Bailer & Kaye, 2011; Steiger, 2004). A substantial body of evidence suggests dysfunctional serotonin signaling (i.e., altered post-synaptic receptor sensitivity, reduced serotonergic neurotransmission) is part of the body of observed neurobiological alterations contributing to ED symptoms (Becker et al., 2022; Madsen et al., 2019; Monteleone et al., 2000). Furthermore, EDs are associated with clinical response to serotonergic medications, with selective serotonin reuptake inhibitors being a commonly prescribed pharmacotherapy for EDs and, in the case of fluoxetine, the only approved pharmacotherapy for BN (Davis & Attia, 2017; Fluoxetine Bulimia Nervosa Collaborative Study Group, 1992; Rodan et al., 2023). It is therefore reasonable to deduce that the serotonergic effects of psilocybin may similarly be beneficial to ED clinical outcomes by normalizing neurotransmitter signaling in serotonergic pathways. Although no study has empirically assessed the relationship between psilocybin-induced serotonergic signaling and clinical outcomes in EDs, the serotonergic effects of psilocybin have been shown to correlate with greater acute psychoactive effects, the latter of which reliably predicts the magnitude of depressive symptom reduction (Levin et al., 2024; Madsen et al., 2019; Roseman et al., 2017; Stenbæk et al., 2021).

#### 3.1.2. Neuroplasticity

Psilocybin is included in the class of pharmacotherapies termed psychoplastogens, a group of therapeutics that rapidly promote structural and functional neuroplasticity (Olson, 2018). Neuroplasticity is a fundamental process for the adaptation and improvement of neural, psychological and behavioral processes, defined as the brain’s ability to change in response to internal and external stimuli. Psilocybin has been shown to stimulate neuroplasticity-related processes, including increased dendritic spine growth, synaptogenesis, and elevated levels of brain-derived neurotrophic factor (BDNF) in preclinical and human studies. Similar to many psychiatric conditions, neuroplasticity is reduced in EDs and considered a biomarker of treatment responsiveness (Keeler et al., 2022, 2024; Phillips et al., 2014; Shobeiri et al., 2022; Zhao et al., 2024). For example, individuals with AN have significantly lower levels of BDNF when compared to the general population (Keeler et al., 2022, 2023; Shobeiri et al., 2022). Regarding psychotherapeutic success, experts suggest psychoplastogens should be paired with structured therapy to not only channel neuroplastic changes toward desired outcomes, but also to avoid maladaptive outcomes (Jones, 2025). In a synergistic manner, the transient period (2–4 weeks) of heightened neuroplasticity induced by psilocybin, often termed the ‘psychedelic after-glow,’ is hypothesized to potentiate the cognitive and behavioral patterns introduced by CBT-ED, thereby supporting durable therapeutic change (Evens et al., 2023; Jones, 2025; Majić et al., 2015).

#### 3.1.3. Neural Network Connectivity

Beyond receptor-level effects, psilocybin induces alterations in large-scale neural network connectivity, as consistently demonstrated by functional neuroimaging studies (Carhart-Harris et al., 2012; Gattuso et al., 2023; Siegel et al., 2024). Specifically, psilocybin is associated with disrupted functional connectivity in the default mode, salience and executive control networks—all of which are implicated in critical cognition- and emotion-related processes. In EDs, altered functional connectivity among the aforementioned neural networks is often observed and associated with cognitive inflexibility, a hypothesized mediator of poor psychotherapy engagement (Gattuso et al., 2023; Koning et al., 2024; Steward, 2024). For example, BN is associated with reduced neural signaling for prediction errors that are critical for reliably and flexibly updating beliefs and cognitions (Berner et al., 2023; Frank et al., 2011). By disrupting large-scale neural networks, psilocybin may create a state of cognitive flexibility that is conducive to psychological flexibility, behavioral modification and learning—changes that both counteract alterations observed in EDs and are beneficial for engagement in psychotherapy (Carhart-Harris et al., 2012; Carhart-Harris & Friston, 2019; Doss et al., 2021; Rutschmann et al., 2024). In depression, alterations in neural network connectivity have been shown to correlate with clinical outcomes (Daws et al., 2022).

Together, the aforementioned neurobiological effects have been implicated in the transdiagnostic therapeutic potential of PT and provide a constellation of potential mechanistic correlates for the psychological manifestations that will be beneficial for CBT-ED. For example, the serotonergic and antidepressant effects of psilocybin are expected to remove a critical barrier (i.e., mood symptoms) to engagement and CBT-ED, thereby reducing dropout. Alterations to neuroplasticity and neural network connectivity are expected to promote enhanced CBT-ED outcomes by fostering learning and durable cognitive/behavioral change. See Koning et al. (2024) for a supplemental review of the potential neurobiological correlates of PT in EDs (Koning et al., 2024).

### 3.2. Psychological Effects

#### 3.2.1. Body Image

Body image disturbances are considered key factors in the maintenance of EDs, including shape/weight-related concerns, fear of weight gain, and/or self-evaluation unduly influenced by body weight and shape in BN and AN (Fairburn, 2008b; Levinson et al., 2017). Although not considered a core criterion for BED, those who report body weight/shape overvaluation tend to have higher levels of overall ED psychopathology and distress when compared to individuals with BED without body weight/shape overvaluation (Grilo, 2013; Lydecker et al., 2017). As such, body image disturbance is considered a major psychological barrier to positive CBT-ED outcomes and often predicts symptom improvement (Calugi & Dalle Grave, 2019; Vall & Wade, 2015). PT is associated with rapid and sustained changes in maladaptive thoughts and cognitions, providing an opportunity to shift distorted and rigid high-level priors that uphold body image disturbances (Carhart-Harris & Friston, 2019; Ho et al., 2020). Calder et al. (2023) provide a more in-depth discussion of the proposed mechanisms in which PT may help overcome maladaptive cognitions related to body image in EDs via altered interoceptive awareness, reduced self-referential processing, and increased self-acceptance (Calder et al., 2023). The potential of PT to counteract body image disturbance is supported by previous PT trials in EDs, including the reorganization of values in AN and reduced body image preoccupation in body dysmorphic disorder (Peck et al., 2023; Zhu et al., 2025). For example, in a phase 1 trial of PT for AN, 60% of participants felt as though the importance they placed on their physical appearance had decreased posttreatment (Peck et al., 2023). These changes align with the established therapeutic goals of CBT-ED and may help accelerate therapeutic change in this domain (Askew et al., 2020; Fairburn, 2008a; Grilo et al., 2009).

#### 3.2.2. Psychiatric Comorbidities

Emotional dysregulation, and depressive and anxiety symptoms represent common comorbidities in EDs and significant barriers to psychotherapy success. For example, EDs carry high lifetime rates of depression (76.3% for BN, 65.5% for BED, and 49.5% for AN) and approximately two-thirds of patients will meet the criteria for an anxiety disorder (Attia & Walsh, 2025; Godart et al., 2003; Kaye et al., 2004; Swinbourne et al., 2012). Depressive symptoms are a well-documented predictor of poor engagement and dropout in CBT-ED (Coker et al., 1993; Schnicker et al., 2013; Steel et al., 2000). Similarly, anxiety (e.g., fear of weight gain) poses a barrier to psychotherapy retention and greater early reductions in anxiety predicts ED symptom improvement post-treatment (Gorrell et al., 2023; Velkoff et al., 2024). Several meta-analyses demonstrate that PT exerts robust antidepressant effects (Fang et al., 2024; Haikazian et al., 2023; Rosenblat et al., 2023) and reduces anxiety, albeit the strongest evidence for anxiolytic effects of PT is in the context of end-of-life distress (Feulner et al., 2023; Irizarry et al., 2022). It is therefore reasonable to hypothesize that PT may help overcome CBT-ED barriers (e.g., dropout and poor engagement) and improve ED outcomes via antidepressant and anxiolytic effects. As reviewed by Calder et al. (2023), the mechanisms of PT also demonstrate potential to improve symptoms of other common comorbidities with EDs, including posttraumatic stress disorder, substance use disorder, and obsessive–compulsive disorder which further complicate and challenge CBT-ED (Calder et al., 2023).

#### 3.2.3. Social Functioning

Social dysfunction is often observed in EDs and correlates with symptom onset, severity, and treatment response (Agras et al., 2000; Keller et al., 1992; Lie et al., 2019; Raykos et al., 2017). Difficulties in social functioning in EDs may manifest as interpersonal avoidance, impaired social reward processing, heightened sensitivity to social evaluation, and challenges building and maintaining relationships (Cardi et al., 2018). Such deficits are not only detrimental to overall quality of life and daily functioning in EDs but may also challenge CBT-ED engagement and success by compromising the therapeutic alliance which is among the strongest predictors of positive outcomes (Graves et al., 2017). Psychedelics have been shown to stimulate oxytocin release—a neuropeptide involved in prosocial behavior—and increase synaptic plasticity in neural circuits responsible for social reward learning (Holze et al., 2021a, 2021b). These changes may improve general social functioning and strengthen the therapeutic alliance, which is hypothesized to promote CBT-ED success (Accurso et al., 2015; Graves et al., 2017).

#### 3.2.4. General Well-Being

Beyond improved relations with others, transdiagnostic empirical and theoretical evidence suggests that PT may help individuals improve their self-relationship and promote general well-being that may be conducive to positive CBT-ED outcomes. EDs frequently involve negative self-appraisals, shame, and experiential avoidance—factors that pose a significant challenge to the cognitive restructuring and self-acceptance goals of CBT-ED (Marques et al., 2021; Nechita et al., 2021; Scott et al., 2014; Waller & Beard, 2024). PT increases emotional processing, openness, and positive changes in subjective well-being in clinical and non-clinical populations (MacLean et al., 2011; Mertens et al., 2020; Wiepking et al., 2023). Participants commonly report sustained increases in self-acceptance, self-compassion, connectedness to values, and perceived insight into the root cause of one’s psychological issues (Crowe et al., 2023; Fauvel et al., 2023; Watts et al., 2017). These changes have been effectively communicated via qualitative analyses, including in a previous PT trial for AN (Breeksema et al., 2024; Crowe et al., 2023; Cuerva et al., 2024; Peck et al., 2025). A recent large-scale survey of prescription and non-prescription drug use in individuals with EDs (*N* = 5123) found that psilocybin was among the highest self-rated drugs for, not only ED symptom improvement, but also overall mental health (Rodan et al., 2025). Together, these general salutary effects are hypothesized to target barriers to psychotherapeutic engagement and success in EDs, including experiential avoidance, self-criticism and shame (Erritzoe et al., 2019; Mertens et al., 2020; Nechita et al., 2021; Sønderland et al., 2024; Wiepking et al., 2023). Moreover, improvements in general well-being may support broader recovery trajectories, including fostering healthier lifestyle behaviors, self-efficacy and improved quality of life which may be protective against relapse (Heal-Cohen et al., 2025).

In most cases, PT elicits the changes described above in 1–2 dosing sessions, suggesting an opportunity to accelerate therapeutic progress and strengthen engagement with the addition of PT to the CBT-ED protocol. This may be especially beneficial considering that early improvement is the strongest predictor of positive CBT-ED outcomes (Dixon et al., 2025; MacDonald & Trottier, 2019).

## 4. Considerations for PT/CBT-ED Protocol Development

The proposed protocol for a combined PT and CBT-ED intervention has been developed based on the hypothetical premise that it will have synergistic therapeutic effects for individuals with EDs. While the intervention primarily focuses on CBT-ED for non-underweight EDs, potential applications to CBT for anorexia nervosa (CBT-AN) are mentioned in the Section 5. This intervention is not intended for current clinical use but is designed to investigate its therapeutic efficacy in future clinical studies.

**Figure 1:**
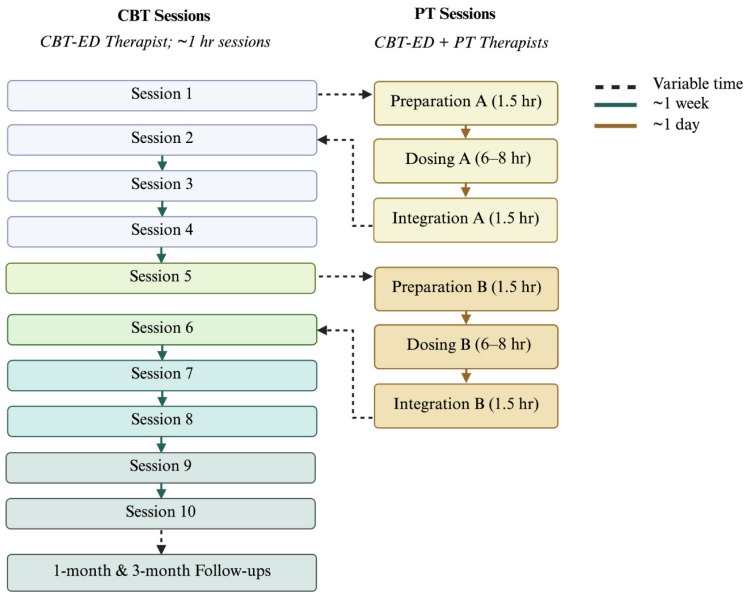
Overview of the timeline for psilocybin treatment as an adjunct to cognitive-behavioral therapy for eating disorders.

The CBT-ED component consists of a structured, time-limited adaptation specifically designed to address core ED symptoms, including binge eating, purging, and associated cognitive distortions. Brief CBT-ED, also known as CBT-Ten or CBT-T, is an evidence-based psychotherapy model for non-underweight EDs, comprising ten sessions followed by follow-up at one and three months (Keegan et al., 2022; Paphiti & Newman, 2023). The proposed intervention involves a standard course of CBT-T augmented by two adjunctive PT sessions, scheduled after the first and fifth CBT-T sessions (see Figure 1). These time points are critical, as outcome data indicate high dropout risks after session one—when rapid, anxiety-provoking behavioral changes are introduced—and after session five, when challenging body image work begins (Dixon et al., 2025).

The PT sessions follow traditional psychedelic therapy practices, structured as a three-step model involving preparation, dosing, and integration. The preparation session is a 90 min session in a private therapy room the day before dosing, involving the CBT-T therapist and one PT therapist who will attend the dosing session. Its aims include building rapport, setting therapeutic intentions, establishing boundaries for nondirective emotional support, gathering participant history, assessing readiness, providing education on psilocybin’s psychoactive effects (including potential adverse physical and psychological impacts), and explaining dosing logistics and safety measures.

The dosing session occurs in a controlled setting with the CBT-T therapist and PT therapist present, lasting up to eight hours, during which participants listen to ambient instrumental music (with input on the playlist) and are monitored for safety before discharge. The environment is private and quiet, free from interruptions, with comfortable seating or lying areas, optional eye masks and headphones, blankets, temperature control, aesthetic appeal, access to food, drink, and bathrooms, locked windows, stereo equipment, video-recording tools, medical supplies, and secure storage for records and investigational products. Aims for this session focus on cultivating an optimal “set” (i.e., mindset, intentions, expectations) and “setting” (i.e., physical space, therapists, music), offering nondirective support with a “beginner’s mind” (i.e., approaching internal experience with openness, curiosity, and nonjudgment; allowing thoughts, emotions, and sensations to arise without preconceived expectations or attempts to control the experience), addressing any resistance through processing rather than avoidance, and ensuring safety and tolerability.

The integration session is a 90 min follow-up the day after dosing, held in a private therapy room with the CBT-T therapist and PT therapist. Its aims involve assessing the participant’s tolerance of PT and linking therapeutic insights to CBT-T concepts, reviewing and processing the dosing experience, including emotional distress, cognitive dilemmas, and affirming insights, and anchoring lessons into daily life to actualize behavioral changes for ED recovery. This includes recognizing the evolving nature of insights, encouraging ongoing support connections, and acknowledging that others (e.g., family members) may not fully grasp the experience’s depth.

For a session-by-session summary of the CBT-T protocol, including integration considerations with PT, refer to Table 2.

## 5. Discussion

### 5.1. Implications

The present article consolidates the theoretical and empirical foundations supporting PT as an adjunct to CBT-ED and proposes considerations for protocol development. The hypothesis that the neurobiological and psychological mechanisms of PT will improve CBT-ED outcomes should be tested in future interventional trials of feasibility, acceptability, tolerability, and preliminary efficacy (e.g., improved ED symptoms and reduced CBT-ED dropout). Our research group plans to do this, although broader use of the protocol considerations in the development of population-specific PT protocols is encouraged. For example, although the protocol outlined above is specific to CBT for non-underweight EDs, a similar approach could be applied to CBT for AN with adaptations to address the unique needs of this population (e.g., ego-syntonic features, weight regain, extended treatment duration). If empirically supported, PT/CBT-ED could address the critical challenge of dropout and poor outcomes that limit therapeutic success from first-line psychotherapies for EDs. 

More broadly, the proposed intervention contributes to the advancement of conventional psychedelic therapy models which are largely based on traditional approaches that include non-directive, non-specific psychological support; these psychotherapy components are often inadequately defined and emphasize concepts of the “inner-healer.” The present framework departs from this historical model by embedding a structured, evidence-based psychotherapy (i.e., CBT-ED) both as concurrent sessions and throughout PT integration. Upon further development of the treatment manual, this shift towards protocol-based psychotherapy may help address longstanding methodological criticisms of psychedelic treatments such as poor fidelity and replicability, and difficulty isolating drug effects from psychotherapy effects. From a clinical perspective, the incorporation of structured psychotherapy may promote more durable behavioral change, as described by the rationale presented above.

### 5.2. Limitations

The major limitation of the interventional framework presented herein is the lack of empirical evidence testing PT-assisted psychotherapies in EDs and the regulatory barriers that continue to hinder this line of inquiry (e.g., psilocybin is a Schedule III controlled substance in Canada). Currently, there are no placebo-controlled trials of PT combined with CBT-ED, and only a handful of small, open-label studies have examined PT in ED populations. Another criticism of PT is the resource-intensive nature of the intervention, including specialized provider training, medical supervision, and drug costs. However, recent economic modeling suggests potential long-term cost savings via improved patient outcomes; for example, Avanceña et al. (2025) found that PT had a 75% probability of being more cost-effective than standard of care for treatment-resistant depression (Avanceña et al., 2025).

### 5.3. Safety & Tolerability Considerations

Psilocybin exhibits a favorable acute safety profile when administered at standard doses (up to 30 mg) in medically supervised settings (Fang et al., 2024; Freitas et al., 2025; Hinkle et al., 2024). The most common (17–40% of cases) side effects are typically mild and transient including nausea, dizziness, fatigue, anxiety, sympathomimetic effects and headache (Hinkle et al., 2024). However, patients with EDs may present unique medical and psychological vulnerabilities such as electrolyte imbalances, cardiovascular abnormalities (e.g., bradycardia, arrhythmias), and possible distress from vomiting/nausea or body image distortions during the dosing session (Downey et al., 2024; Lacroix et al., 2024). These risks warrant careful screening prior to participation in PT, as well as medical monitoring during dosing to manage adverse reactions and provide psychological support throughout challenging experiences. In a PT trial in AN, no serious adverse events or significant changes in vital signs occurred and the intervention was deemed safe and tolerable (Peck et al., 2023).

### 5.4. Research Recommendations

Moving forward, several publications call for increased funding for psychedelic studies in EDs (Bevione et al., 2025; Otterman, 2023; Xi et al., 2023) and studies which examine PT as an add-on to evidence-based psychotherapy (Cuerva et al., 2024; Goodwin et al., 2024; Wolff et al., 2025). Pilot trials are needed to establish feasibility, optimal dosing, acceptability, and preliminary efficacy of PT/CBT-ED. Subsequent phase II randomized, double-blind, placebo-controlled trials should incorporate active comparator arms to address functional unblinding and expectancy bias which remain major methodological limitations in psychedelic research (van Elk & Fried, 2023). The mechanisms proposed above may be investigated via neurobiological (e.g., neuroimaging, plasma neurotrophin analysis) and phenomenological approaches in order to extract insights about precise therapeutic actions (Koning et al., 2025). If there is sufficient evidence of therapeutic efficacy, larger trials should examine durability of intervention outcomes, long-term safety, and cost-effectiveness as appropriate. Increased public and private funding, expanded investigator training programs, and regulatory facilitation will be critical to realizing this research agenda.

## 6. Conclusions

Together, this narrative review presents evidence to support the therapeutic potential of a combined PT/CBT-ED intervention—a potential solution to the critical problem of poor psychotherapy outcomes and high dropout in EDs. Considerations for a concurrent intervention framework are also provided to facilitate future research initiatives evaluating the utility of PT to enhance evidence-based psychotherapy models. This line of inquiry is expected to contribute to the development of innovative approaches that improve ED treatment and, more broadly, advance the study of psychedelics as tools to enhance evidence-based psychotherapy models. 

## Figures and Tables

**Table 1:** A summary of the hypothesized mechanisms in which psilocybin treatment may augment cognitive-behavioral therapy for eating disorders.

Mechanism	ED Psychopathology and CBT-ED Barriers	Psilocybin Treatment
Neurobiological		
Serotonergic neurotransmission	Dysregulated serotonin signaling; clinical response from serotonergic medications	↑ serotonin signaling
Neuroplasticity	↓ markers of neuroplasticity	↑ markers of neuroplasticity
Neural network connectivity	Maladaptive neural network connectivity; ↓ cognitive flexibility	Disrupted functional connectivity; ↑ cognitive flexibility
Psychological		
Body image	↑ body image overvaluation	↓ body image overvaluation
Mood	↑ depressive and anxiety symptoms	↓ depressive and anxiety symptoms
Social function	Social dysfunction; challenges to therapeutic alliance	Prosocial effects; strengthened therapeutic alliance
General well-being	Experiential avoidance, shame, self-criticism, low self-esteem	Self-acceptance/compassion, sense of connectedness and insight

Note: ‘↑’ and ‘↓’ represent increased and decreased, respectively.

**Table 2:** Session-by-session CBT-T program with considerations for psilocybin treatment integration.

Phase	Session	Key Activities & Topics	PT Considerations/Facilitator Guidance
**Phase 1: Early engagement & eating structure**	1	Introduce CBT-T model and describe phases Review current eating and begin food monitoring Introduce ‘Energy Graph’ Introduce rationale for weighing and take weights Review timing of the integration of PT	Discuss the potential impact of the dosing session on appetite and how to work on normalizing eating structure within this framework. PT-induced emotional openness might help patients reduce emotional distress and experiential avoidance, increasing willingness to engage in early CBT-T tasks such as food monitoring, regular eating, and weighing; processes that often precipitate early dropout. PT session explicitly linking PT-derived insights (e.g., values clarification, reduced shame, altered self-narratives) to CBT-T concepts may help translate experiential learning into concrete behavioral commitments within ongoing CBT-T sessions. Discuss potential for antidepressant/antianxiety effects of the PT session. Patients should lean into openness to make changes as soon as possible given the influence of early behavior change on ED outcomes. Prosocial and affiliative effects of PT may enhance trust, emotional disclosure, and collaboration with the CBT-T therapist, facilitating a stronger therapeutic alliance, an established predictor of CBT-T retention and outcome. May introduce emotional triggers content earlier for patients who notice changes in self-acceptance, self-concept, and flexibility.
	2	Briefly review the first PT experience Review food records and develop regular eating plan	
	3	Review food records and develop regular eating plan Introduce feared/safe food List or behavioral experiment for fast-progressing patients	
	4	Review progress and decide to continue (or not) Build and execute experiments with feared foods	
**Phase 2: Behavioral Experiments**	5	Build and execute experiments with feared foods Introduce Newton’s Cradle analogy and extended food records	PT-associated increases in psychological flexibility and openness may reduce rigid, rule-governed eating behaviors, allowing patients to approach feared foods and behavior experiments with greater tolerance of uncertainty and distress. Prepare the patient for a second PT session. Explore potential impact on the rest of treatment. For example, how improved mood will influence emotional triggers and how self-acceptance will aid body image work.
**Phase 3: Emotional Triggers** **(Behavioral Experiments if needed)**	6	Explore core beliefs and links between early experiences and current beliefs Introduce checking or comparison body image experiment	The second PT after Session 5 could mitigate experiential avoidance in body image exercises, such as mirror exposure; patients might draw on PT experiences of altered body perception to process distress, resulting in deeper therapeutic engagement and improved outcomes in subsequent sessions focused on shape/weight concerns. Increased emotional openness and access to autobiographical material following PT may support more effective exploration of emotional triggers, core beliefs, and early learning experiences within CBT-T emotional processing modules.
**Phase 4: Body Image** **(Behavioral Experiments and/or Emotional Triggers if needed)**	7	Review body checking or comparison homework Explain imagery rescripting	PT-associated increases in self-acceptance, self-compassion, and reduced body image overvaluation may attenuate resistance to body-focused interventions, a phase of CBT-T commonly associated with dropout and stalled progress. If significant changes in self-love, self-acceptance, and self-concept have transpired, may be able to shorten body image content.
	8	Complete imagery rescripting exercise to modify negative core beliefs and develop self-compassion Introduce mirror exposure	
**Phase 5: Relapse Prevention** **(Body image if needed)**	9	Mirror exposure and/or mind reading survey Introduce ‘therapy blueprint’ to develop relapse prevention plan	Transient increases in neuroplasticity following PT may potentiate the acquisition and consolidation of CBT-T skills (e.g., flexible thinking, adaptive coping, relapse prevention strategies), leading to more durable cognitive and behavioral change.
	10	Finalize ‘therapy blueprint’ Discuss self-monitoring post-therapy Highlight progress and reinforce use of learned strategies Encourage autonomy and confidence in managing future challenges.	
**Follow-up Phase**	11 12	Review Therapy Blueprint Problem-solve any challenges and/or provide positive reinforcement	PT-fostered “beginner’s mind” approach might encourage patients to view setbacks as learning opportunities, enhancing long-term behavioral maintenance during follow-up at 1 and 3 months.

## Data Availability

The original contributions presented in this study are included in the article. Further inquiries can be directed to the corresponding author.

## Abbreviations

The following abbreviations are used in this manuscript:

ED	Eating Disorder
AN	Anorexia Nervosa
BN	Bulimia Nervosa
BED	Binge eating disorder
CBT	Cognitive-behavioral therapy
PT	Psilocybin treatment
MAPS	Multidisciplinary Association for Psychedelic Studies
MDMA	3,4-methylenedioxymethamphetamine
BDNF	Brain-derived neurotrophic factor
